# MultiBac: Baculovirus-Mediated Multigene DNA Cargo Delivery in Insect and Mammalian Cells

**DOI:** 10.3390/v11030198

**Published:** 2019-02-26

**Authors:** Kapil Gupta, Christine Tölzer, Duygu Sari-Ak, Daniel J. Fitzgerald, Christiane Schaffitzel, Imre Berger

**Affiliations:** 1 School of Biochemistry, Biomedical Sciences, University of Bristol, 1 Tankard’s Close, Bristol BS8 1TD, UK; kapil.gupta@bristol.ac.uk (K.G.); ct17870@bristol.ac.uk (C.T.); christiane.berger-schaffitzel@bristol.ac.uk (C.S.); 2 Bristol Synthetic Biology Centre BrisSynBio, University of Bristol, 4 Tyndall Ave, Bristol BS8 1TQ, UK; 3 European Molecular Biology Laboratory EMBL, 71 Avenue des Martyrs, 38000 Grenoble, France; duygusari05@gmail.com; 4 Geneva Biotech SARL, 64 Avenue de la Roseraie, 1205 Genève, Switzerland; df@geneva-biotech.com

**Keywords:** baculovirus, heterologous expression, multiprotein complex, human TFIID, GPCR, virus-like particle VLP, transduction, drug screening, genome engineering, synthetic biology

## Abstract

The baculovirus/insect cell system (BICS) is widely used in academia and industry to produce eukaryotic proteins for many applications, ranging from structure analysis to drug screening and the provision of protein biologics and therapeutics. Multi-protein complexes have emerged as vital catalysts of cellular function. In order to unlock the structure and mechanism of these essential molecular machines and decipher their function, we developed MultiBac, a BICS particularly tailored for heterologous multigene transfer and multi-protein complex production. Baculovirus is unique among common viral vectors in its capacity to accommodate very large quantities of heterologous DNA and to faithfully deliver this cargo to a host cell of choice. We exploited this beneficial feature to outfit insect cells with synthetic DNA circuitry conferring new functionality during heterologous protein expression, and developing customized MultiBac baculovirus variants in the process. By altering its tropism, recombinant baculovirions can be used for the highly efficient delivery of a customized DNA cargo in mammalian cells and tissues. Current advances in synthetic biology greatly facilitate the construction or recombinant baculoviral genomes for gene editing and genome engineering, mediated by a MultiBac baculovirus tailored to this purpose. Here, recent developments and exploits of the MultiBac system are presented and discussed.

## 1. Introduction

In 1983, Max Summers and his team reported the successful production of a heterologous protein, human IFN-β, in insect cells by using a recombinant baculovirus [1]. They had modified the genome of *Autographa californica multiple nuclear polyhedrosis virus* (AcMNPV) and transfected insect cell cultures derived from the fall armyworm with the recombinant AcMNPV genome. Previously, they had realized that a viral protein, polyhedrin, is expressed at very high levels very late in the viral life cycle but was dispensable for maintaining an infectious virus in laboratory culture. By replacing the polyhedrin gene with their heterologous gene of choice, Summers and his team could exploit the machinery of the virus to drive the high-level expression of IFN-β which they could then purify [2]. This remarkable feat demonstrated the utility of baculovirus for heterologous protein production, and the baculovirus/insect cell system (BICS) has since been used to produce many proteins, accelerating research and development in laboratories world-wide, in academia and industry.

Originally used for recombinant protein production, the scope of the technology experienced significant expansion with the discovery that the tropism of the virus could be altered to transduce mammalian cells, acting as a DNA delivery tool [3,4,5]. If genes were now placed under mammalian active promoters, baculovirus could be manufactured in insect cells and then administered to mammalian cells and even tissues or organisms to realize this particular genetic information, introducing baculovirus as a gene therapy vector. These developments have been authoritatively reviewed in numerous publications including this Special Issue in *Viruses* [2,6,7,8,9,10,11]. We will thus focus here on our own contributions to baculovirus technology, the MultiBac system, which we introduced some fifteen years ago [12,13,14]. We had the privilege over the years to contribute periodical reviews about MultiBac developments and—to our delight—its increasingly widespread adoption in the research community [13,15,16,17,18,19,20,21,22,23,24]. To forestall boring the audience, we will therefore restrict ourselves to just briefly summarizing the essentials of MultiBac, and then proceed to highlight in the present review the most recent exploits and developments, by us and others, of this baculoviral system we have conceived.

## 2. The MultiBac BICS: Enabling Multiprotein Complex Structure Analysis

Our incentive to develop MultiBac originated from the emerging realization that proteins in cells rarely function in isolation, but often accommodate in supramolecular assemblies with several to many other proteins and other biomolecules to carry out their chores [25]. Elucidating how these ensembles work at a molecular resolution necessitates methods to produce and purify them in the quality and quantity required for structural and mechanistic studies. Some assemblies such as ribosomes or proteasomes are prevalent in cells and can be purified from native source material. Many others however are characterized by a low abundance requiring recombinant overexpression. Recombinant baculovirus can express heterologous proteins at high levels. Further, it uses eukaryotic insect cell culture as a host and can thus provide authentic post-translational modification which may be important for the integrity and activity of a specimen studied. Furthermore, the narrow host range of baculovirus requires only standard laboratory safety provisions. All these were good reasons to choose baculovirus as the system to produce those essential molecular machines.

**Figure 1:**
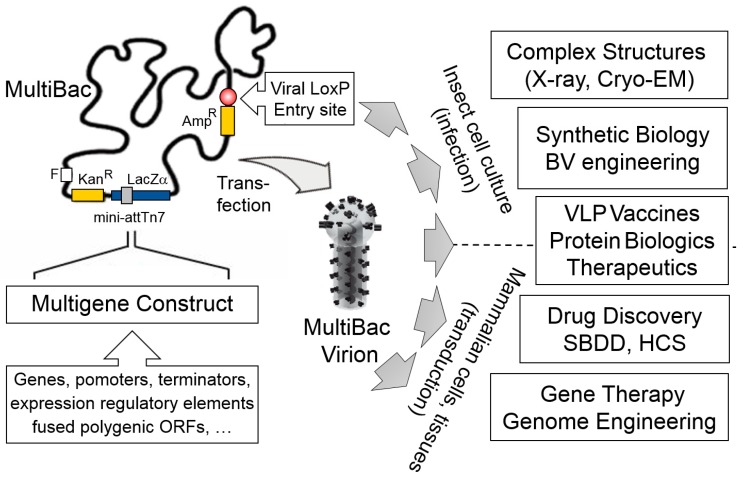
The MultiBac system. MultiBac consists of an engineered baculoviral genome optimized for multigene delivery and protein complex expression (left). The genome is present as a bacterial artificial chromosome (BAC) in *E. coli* cells supplying the Tn7 transposase. Expression cassettes comprising genes of interest and gene regulatory elements are assembled into the multi-gene expression constructs and inserted into the MultiBac genome by Tn7 transposition. The Tn7 attachment site is embedded in a LacZα gene enabling blue-white screening of recombinants. A second entry option into the viral backbone is provided distal from the Tn7 site, relying on Cre recombinase catalysed site-specific integration into a LoxP sequence on the viral backbone (circle filled in red). This site can be used to customize MultiBac by providing additional functionalities. Composite MultiBac baculoviral DNA containing all DNA elements of choice is extracted from *E. coli* cultures, followed by transfection into insect cell cultures to manufacture functional MultiBac virions. These are then used for a wide range of applications (right), by the infection of insect cell cultures or transduction of mammalian cells, tissues and organisms. Cryo-EM, electron cryo-microscopy; X-ray, crystallography; BV, baculovirus; VLP, virus-like particle; SBDD, structure-based drug design; HCS, high-content screening. Amp, ampicillin; Kan, kanamycin; F, bacterial F replicon; LacZα, gene encoding B-galactosidase α fragment; mini-attTn7, minimal Tn7 attachment site. Virion image is adapted from drawing kindly provided by Kari Airenne [28].

Baculovirus/insect cell systems were already available back then including tool-kits which conveniently utilized a baculoviral genome in the form of a bacterial artificial chromosome (BAC), originally developed by Luckow and colleagues [26]. This BAC is propagated in *E. coli* cells and relies on Tn7 transposase mediated integration of the foreign gene of interest from a transfer plasmid into the BAC [27]. This setup allowed us to reengineer the baculoviral genome in a rather straight forward fashion in *E. coli*, eliminating undesired functions such as viral proteolytic and apoptotic activities and adding advantageous modalities such as a site-specific recombination sequence (LoxP) to integrate additional foreign genes distal from the Tn7 attachment site (Figure 1) [14,21].

To facilitate the assembly of multi-gene expression cassettes, we developed recombineering-based, parallelized, automatable approaches relying on synthetic DNA plasmid modules that could be conjoined into elaborate transfer plasmids for integration into the Tn7 and LoxP sites, respectively [29,30,31,32]. We followed a ‘reduce to the max’ approach in designing these reagents, eliminating from our plasmid modules DNA elements with unclear or, for our purposes unnecessary, functions present in common plasmids, keeping only the bare minimum of DNA elements conferring defined functions (origin, resistance marker, promoters and terminators), adding the specific integration sequences we needed (LoxP, Tn7) and short DNA sequences for multiplying the expression cassettes. This approach yielded a significantly streamlined, functional tool-box of DNA substantially simplifying the construction of multifunctional recombinant baculovirus specimens which could be customized with ease [16,24,33]. More recently we have extended this reductionist approach also to the baculoviral genome itself, a considerable fraction of which appears to be dispensable for, or even detrimental to, maintaining and deploying it in the laboratory [19,23]. Of note, all these developments greatly profit from the remarkable ongoing reduction in cost of custom DNA synthesis. We anticipate that in the near future, the cross-over may be reached to render commercial DNA synthesis a *bona fide* economic alternative even to the streamlined current assembly approaches for constructing multigene expression cassettes, which we and others have put in place [31,32,34,35].

**Figure 2:**
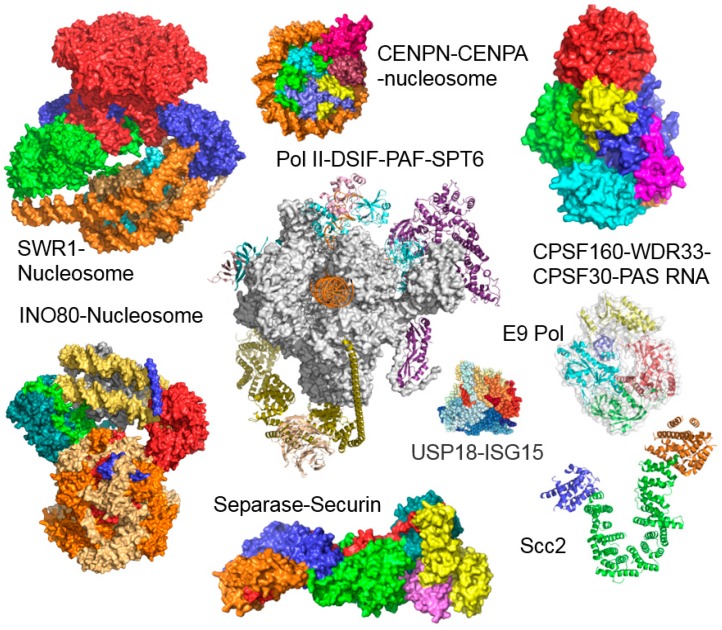
Multiprotein complex structures. Chromatin remodeling enzymes SWR1 (14 subunits) and INO80 (11 subunits) were produced by using MultiBac, bound to nucleosomes and the molecular structures determined by electron cryo-microscopy [37,38,39,40,41,42]. The yeast polII DSIF-PAF-Spt6 cryo-EM structure utilized RNA polymerase II from native source reconstituted with recombinantly expressed associated proteins [43]. Further recent structures of sample produced with the MultiBac BICS include the CENPN-CENPA-nucleosome complex [44], the human CPSF-160–WDR33–CPSF-30–PAS RNA quaternary complex [45], the E9 polymerase [46], the USP18-ISG15 complex [47], the Separase-Securin complex [48] and the cohesion loader Scc [49].

In addition to developing the reagents, we worked out detailed, simple and user-friendly protocols for the multi-gene expression cassette assembly, composite baculovirus generation, insect cell culture maintenance as well as virus amplification and protein complex production. A particularly useful asset was the incorporation of a gene encoding yellow fluorescent protein (YFP) into the backbone of our engineered MultiBac baculovirus. This enables users to follow virus performance and heterologous protein complex production simply by tracking the YFP signal, providing a quantitative means to assess virus quality and protein product quantity, as well as variability from batch to batch. We validated our system and communicated the MultiBac reagents and protocols we developed which were immediately well received by the community, underscoring the existing need for heterologous expression systems that could enable the high quality production of large multi-subunit protein assemblies. The superior performance of our MultiBac system, compared to other existing baculoviral systems, was compellingly demonstrated recently in an elaborate study undertaken by the network of protein production core facilities in Europe (P4EU) [36]. In the meantime, a large and rapidly growing number of multiprotein complexes have now been produced, by us and many others, unlocking their exciting structures and mechanisms at high resolution by X-ray crystallography and electron cryo-microscopy (cryo-EM), resulting in a host of high profile publications. A by no means exhaustive selection of impressive structures reported only in the short time span since our last MultiBac review in 2017 [19] is depicted in Figure 2, exemplifying the fast pace of the field, enabled in part by the baculoviral multiprotein complex production tool-kit we contributed.

## 3. Mechanisms of Transcription Factor Complex Assembly

**Figure 3:**
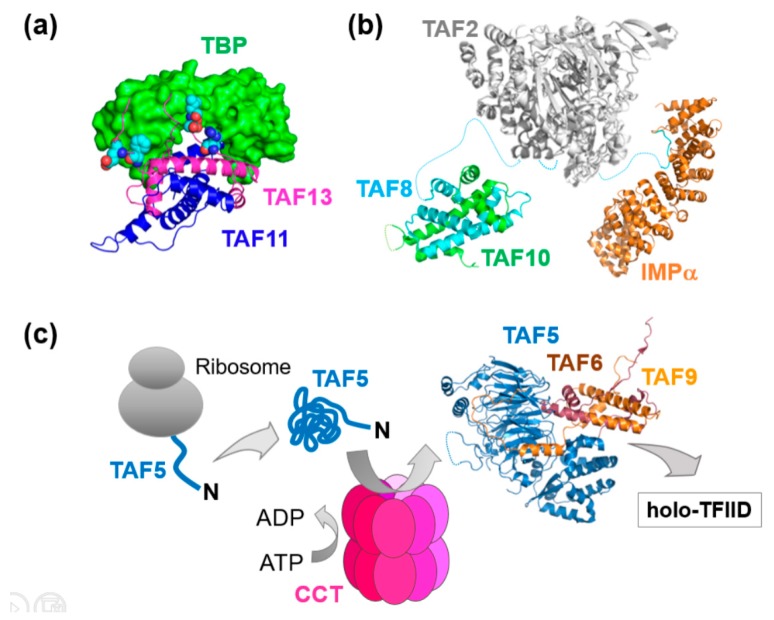
Partial TFIID complexes and mechanisms of TFIID assembly. (**a**) The architecture of TAF11/TAF13/TBP revealed an unexpected binding mode with the TAFs invading the DNA binding groove of TBP [53]. A MultiBac baculovirus customized for genetic code expansion, MultiBac-TAG, was utilized to probe the interaction interfaces by inserting UV-activatable amino acids for cross-linking [55]. (**b**) A nuclear import particle (NIP) formed by TAF8, TAF10, TAF2 and importin is shown, reconstituted from recombinant TAF proteins co-expressed by MultiBac and Importin produced in bacteria [54]. The structures of TAF8/TAF10 and TAF8/Importin were determined by X-ray crystallography, a homology model of TAF2 is shown [50]. (**c**) X-ray crystallography combined with mutational studies, proteomics and cell-based experiments revealed a crucial checkpoint function of the adenylate-dependent chaperonin CCT in the formation of a TAF5/TAF6/TAF9 complex in early stages of human TFIID assembly. CCT captures nascent TAF5 as it emerges from the ribosome, and hands-over folded TAF5 to a preformed TAF6/TAF9 complex in the cytoplasm [51].

The impetus for our own team in putting the MultiBac system in place was derived from our desire to unlock the structure, mechanism and cellular assembly of key multiprotein complexes in human gene expression, with specific focus on human general transcription factors (GTFs) and the pre-initiation complex (PIC) [50]. A central cornerstone of this elaborate machinery of more than a hundred proteins is GTF TFIID, a megadalton-sized multiprotein complex comprising about 20 subunits made up of the TATA-box binding protein (TBP) and its associated factors (TAFs) [50]. TFIID is the first factor to bind the core promoter, thereby nucleating PIC formation. Notably the discovery of distinct TFIID complexes, some of them confined to specific tissues, with different subunit composition, and the observation that several TFIID subunits are shared with other regulatory complexes, imply a dedicated assembly mechanism at work in cells [50]. The underlying mechanisms, however, remain poorly understood. We are addressing these questions by producing partial and holo-TFIID complexes by using MultiBac, determining structures by hybrid methods and scrutinizing cellular compartments in vivo for functional subassemblies and their specific mechanisms which may shed light on TFIID assembly pathways and the cellular factors involved (Figure 3) [50,51,52,53,54].

We already determined by hybrid methods the architecture of a core-TFIID complex present as a distinct, stable entity, possibly with own, specific gene regulatory functions, in the nuclei of cells [52]. Moreover, we identified hitherto unexpected modalities of TBP binding by two TFIID subunits, TAF11 and TAF13, in vitro and in vivo, hinting at substantial conformational dynamics within TFIID to initiate PIC formation and ultimately transcription, possibly depending on the promoter context (Figure 3a) [53]. In this study, we implemented genetic code expansion by engineering MultiBac-TAG, our customized BICS to probe molecular interfaces by incorporating UV-activatable amino acids at specific positions in the primary sequence of the proteins studied [53,55].

Probing the cytoplasm of HeLa cells, we discovered a nuclear import particle (NIP) formed by TFIID subunits TAF2, TAF8, TAF10 and importin, implicating nuclear transport as a regulatory mechanism for holo-TFIID assembly (Figure 3b) [54]. To balance the stoichiometry of the TAFs produced, we implemented a polyprotein strategy [56]. Here, subunits of a complex are produced from a single open reading frame including the gene for a highly specific protease, which efficiently cleaves at specific sites in between the individual subunits within the polyprotein produced [56,57]. Interestingly, our findings triggered our first collaborative study with clinicians investigating genomic mutations present at the interaction interfaces we had identified within the NIP, that lead to severe mental retardation [58]. This study demonstrated the essential nature of these interactions for holo-TFIID integrity and activity. At the same time, it challenged the prevailing concept that complete holo-TFIID is absolutely required for life. In immortalized fibroblasts from the patient, who clearly is alive, practically no holo-TFIID complex could be identified, it had seemingly fallen apart [58].

Recently, we implemented a state of the art proteomics approach to scrutinize in unprecedented detail the presence of TAFs and TBP in cellular compartments. We found a different partial TFIID complex, formed by TAF5, TAF6 and TAF9, existing as a prevalent, distinct entity in the cytoplasm. In the process, we discovered a critical checkpoint function of a chaperonin, CCT, in catalyzing the assembly of this complex (Figure 3c) [51,59]. This intriguing finding sheds first light on the involvement of a cellular factor in early stages of TFIID assembly and may be paradigmatic for the formation of other multiprotein complexes regulating transcription and gene expression, and other vital activities, in cells.

## 4. G-Protein Coupled Receptor (GPCR) Structure and Mechanism

**Figure 4:**
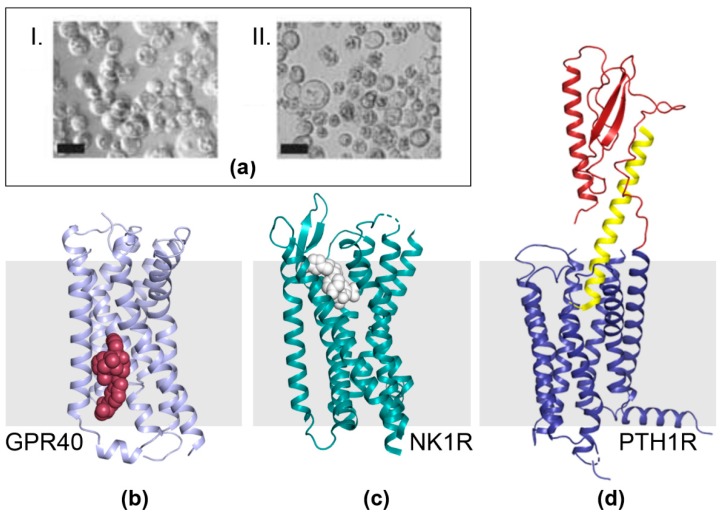
GPCRs made by MultiBac. (**a**) Micrographs show cells 72 h after infection with MultiBac and, for comparison, a commercial competitor baculovirus (Bac-to-Bac, Invitrogen), respectively [12,14]. MultiBac-infected insect cells (I.) are uniformly round and appear intact in contrast to the cell lysis prevalent for Bac-to-Bac infected cells (II.). The elimination of proteolytic and apoptotic factors from the MultiBac backbone significantly improved the integrity of membrane bilayers of infected insect cells at very late stages of the viral life cycle. Scale bars, 20 μm. (**b**–**d**) GPCRs were produced by using the MultiBac BICS and crystallized. X-ray structures of ligand-bound free fatty acid receptor GPR40 (violet) bound to a proprietary ligand (compound 1, red) [70] (**b**), neurokinin 1 receptor NK1R (green) bound to ligand aprepitant (white) [71] (**c**) and parathyroid hormone 1 receptor PTH1R (blue and red) bound to peptide ligand ePTH (yellow) [69] (**d**) are shown. The membrane bilayer is depicted schematically (grey bar).

We observed early on that the alterations we had engineered into the MultiBac baculoviral genome resulted in delayed lysis of cells upon infection with our recombinant virus. Baculovirus takes over the host cell machinery upon infection and either shuts down or reprograms cellular functions to ramp up virus production. Given that many cellular functions terminate, cells upon infection can be looked at as enveloped micro-bioreactor devices rather than ‘living’ cells. Typically, in BICS, heterologous protein expression is driven by viral promoters which are maximally active in the late or very late phase of the viral life cycle in the infected cells, most prominent are the p10 and polh promoters. In wild-type virus, this stage coincides with host liquefaction to release the virus into the environment for infecting new hosts [2,60]. In cell culture, the late and very late phase is characterized by a wide-spread lysis of cells (i.e., the mirco-bioreactors), release of cellular components into the media and accumulation of cell debris (Figure 4a). However, this is also the stage where recombinant protein production is maximal, implying a trade-off between the integrity of the micro-bioreactor device and its capacity to accumulate a heterologous product. This could be particularly relevant for membrane proteins that occupy the cell plasma membrane—it is conceivable that micro-bioreactors with an intact membrane envelope would be advantageous for their production as compared to bio-reactors that become leaky and disintegrate when production of the membrane protein of choice is at its peak.

Among membrane proteins, G-protein coupled receptors (GPCRs) have gained especial prominence. GPCRs are key components of a multitude of signaling cascades. Moreover, they are present on cell plasma membranes and thus more easily ‘reachable’ for modulators as compared to intracellular proteins where one or several membrane bilayer barriers must be navigated before the target is bound. Thus, for many reasons, GPCRs are highly attractive drug targets and their structure determination, bound to agonists, antagonists, small molecules and protein ligands is intensely pursued in the life sciences and pharmaceutical drug development [61,62,63,64,65,66,67,68]. Insect cells appear to be well suited for producing heterologous G-protein coupled receptors, consequently, many GPCRs are being produced by BICS. The MultiBac system we have developed is contributing to this trend, and a selection of recent high-impact ligand-bound GPCR structures, enabled by MultiBac [69,70,71], is depicted below (Figure 4b–d). As hetero-oligomeric GPCRs and GPCRs complexed with accessory proteins come increasingly in focus, we expect the impact of MultiBac to significantly increase in this vibrant research field [72,73].

## 5. VLP-Factory^TM^: Customized MultiBac Baculovirus for Virus-Like Particle Production

**Figure 5:**
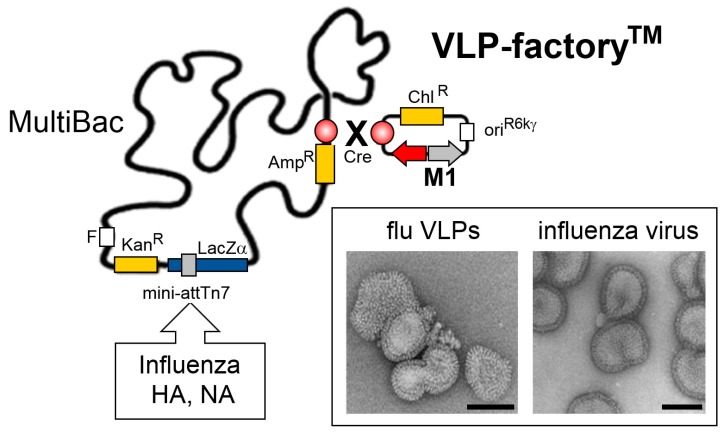
MultiBac-based VLP-factory^TM^. A plasmid module comprising expression cassettes for the capsid-forming influenza H1N1 M1 protein (colored in grey) and a fluorescent protein marker, mCherry (colored in red), was introduced into the MultiBac baculoviral genome by Cre recombinase enzyme mediated plasmid fusion into the LoxP site (circle filled in red, gradient) present on the viral backbone (top right). Genes encoding for hemagglutinin (HA) and neuraminidase (NA) from one or several influenza strains can be integrated into the Tn7 attachment site distal from LoxP. Co-expression of HA, NA and M1 yields synthetic influenza virus-like particles (VLPs) resembling live influenza virus as shown in electron micrographs (boxed) [78]. Scale bars, 100nm. Abbreviations as in Figure 1; ori^R6Kγ^, conditional origin of replication; Chl, chloramphenicol.

Infectious diseases continue to plague populations and economies. Influenza, for example, while rarely deadly, still causes a substantial global economic shortfall every year. In addition, the significant threat of pandemics remains highly acute [74]. Vaccines constitute a premier defense against infectious diseases including influenza [75]. Recombinant virus-like particles (VLPs) which simulate live viruses but lack genetic content and thus are safe, constitute attractive, cost-effective alternatives to inactivated viruses which have dominated the selection of influenza vaccinations to date [76,77]. We recently engineered a version of MultiBac for efficient expression of VLPs based on the influenza M1 capsid protein [78]. We chose M1 from the influenza strain H1N1 as, already by itself, H1N1 M1 produced stable enveloped capsids budding off the infected insect cells in superior quantities as compared to M1 originating from other strains. The gene encoding for H1N1 M1 was inserted into the viral backbone at the LoxP site (Figure 5). A gene encoding a fluorescent protein (mCherry) was supplied along with H1N1 M1 to monitor virus performance and VLP production (Figure 5). This customized MultiBac variant, called VLP-factory^TM^, enabled production of an array of influenza VLPs presenting hemagglutinin (HA) and neuraminidase (NA) proteins from various influenza strains, including HA mutants thought to modulate the host immune system [79]. These influenza VLP variants hold promise to develop into VLP-based hyper-immunogenic antigens that could lead to broadly protecting influenza vaccines by eliciting a strong antibody titre upon immunization in contrast to wild-type HA which by itself is not particularly immunogenic. 

Our VLP-factory^TM^ is not restricted to producing influenza VLPs only. Most viral envelope proteins that can be expressed efficiently in insect cells will be incorporated in M1-based VLPs during the budding process. Thus, many other enveloped VLPs, displaying antigenic proteins from a wide range of viruses, can conceivably be manufactured using our approach, to produce potent VLP-based vaccines to combat infectious disease.

## 6. MultiBacMam-BiFC: Cell-Based Screening by Bimolecular Fluorescence Complementation

The scope of baculovirus-based DNA delivery was substantially expanded by the finding that mammalian cells can be transduced by a recombinant baculovirus and used for heterologous expression if appropriate, mammalian-cell active promoters were provided [3,4,5]. We showed recently that appropriately modified MultiBac baculovirus, MultiBacMam, can be efficiently deployed to deliver complex multifunctional DNA circuitry in mammalian cells and tissues including cell types known to be recalcitrant to transfection by conventional, plasmid-based approaches [19,80]. We used our approach successfully to facilitate gene editing by CRISPR-Cas9 in mammalian cells including primary neurons [80].

**Figure 6:**
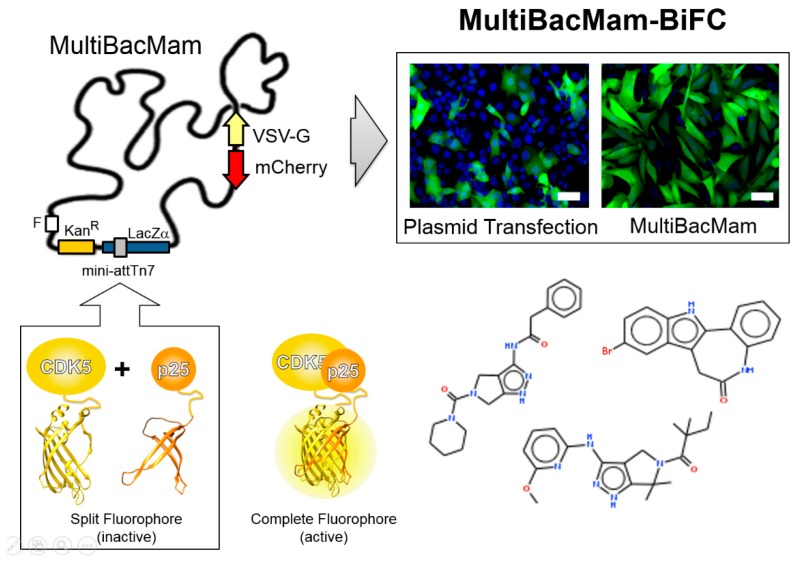
MultiBacMam-BiFC for compound screening. The MultiBacMam baculovirus is shown (top left). Genes encoding for vesicular stomatitis virus glycoprotein (VSV-G) and mCherry to track virus performance during manufacturing have been integrated into the baculoviral backbone, each controlled by baculoviral late promoters. MultiBacMam was outfitted in the Tn7 site with genes encoding for CDK5 and p25, each fused to complementary fragments of a split fluorescent protein, which, upon CDK5-p25 complex formation reconstitute complete, active fluorophore (bottom left). Our setup can be adapted to any PPI. Composite MultiBacMam baculovirus is produced in insect cells and then used to transduce mammalian cells (here U2OS) with superior efficacy as compared to plasmid transfection (top right). BiFC signal is shown in green and Hoechst 33342 nuclear staining in blue. Scale bar, 50 µm. A selection of chemical compounds inhibiting the CDK5-p25 PPI is depicted (bottom right), identified by using our MultiBac-BiFC cell-based screening assay. Abbreviations as in Figure 1.

Building on these results, we have constructed a MultiBacMam variant to assay protein-protein interactions (PPIs) by florescence complementation in living mammalian cells [81]. PPIs are central to the interplay of cellular factors in health and disease, and targeting PPIs, for example by small molecules, is at the forefront of drug discovery. Instrumental to understanding how small molecules can enhance or disrupt PPIs are cellular assays which closely recapitulate native conditions in vivo. These depend on non-invasive, highly sensitive and faithful readout of the PPI and the impact of additives under scrutiny. Bimolecular fluorescence complementation (BiFC) can provide such a readout [82,83,84]. Therefore, we have integrated modalities for BiFC into our engineered MultiBacMam baculovirus (Figure 6). We interfaced our reagents with high-content screening (HCS), resulting in a powerful system for assay development, identification and characterization of small molecule PPI modulators [81]. We implemented our assay to scrutinize chemical compounds modulating the PPI formed by cyclin-dependent kinase 5 (CDK5) and p25, a fragment of the p35 activator. The CDK5-p25 PPI is implicated in many diseases including Alzheimer’s [85,86,87]. With our MultiBacMam-BiFC tool-kit, we implemented cell-based screening to analyse small molecules in a dose-dependent manner and discovered novel compounds which effectively abolished the CDK5-p25 PPI [86].

## 7. Conclusions and Outlook

In 2004, we first communicated our original MultiBac BICS, comprising an engineered baculovirus and a set of reagents specifically tailored to produce multiprotein complexes in the quality and quantity required for high-resolution structural and mechanistic studies. Today, MultiBac is put to excellent use in many laboratories world-wide, in academia and industry, accelerating research and development. Many multiprotein complexes, vital catalysts of cellular function, have been produced and their molecular structures determined, shedding light on their function. The inherent, large heterologous DNA cargo capacity of the baculovirion allowed us and others to insert many genes encoding for the subunits of multiprotein complexes together with synthetic promoters, terminators and diverse gene regulatory elements into recombinant MultiBac baculoviral genomes. In addition to this DNA cargo, a variety of other functions can be provided if needed, including markers to monitor virus performance, insect cell infection levels and the time of harvest when maximal protein production is achieved. Simultaneously, DNA encoded functions can be added including kinases, phosphatases, chaperones and other factors to activate or inactivate a given complex or to assist in proper folding. All this foreign DNA is delivered to insect cell cultures upon infection and translated into elaborate programs yielding high quality complex biological specimens. 

This unprecedented, large DNA cargo capacity also renders baculovirus highly attractive as a tool for DNA delivery in mammalian cell types, for a range of applications including drug discovery, cellular reprogramming and genome engineering, some of which we have touched upon here. We anticipate that the emergence of innovative methods in synthetic biology will enable cost-effective crafting of new and powerful designer baculoviral genomes from scratch, tailored to deploy multifunctional DNA circuitry faithfully in cells, tissues and even organisms, translating encoded messages into elaborate programs to carry out a multitude of tasks, accelerating discovery.
